# Editorial: Neuroimaging of the aging brain

**DOI:** 10.3389/fnimg.2025.1724972

**Published:** 2025-11-14

**Authors:** Owen T. Carmichael, Danielle Harvey, Evan Fletcher

**Affiliations:** 1 Biomedical Imaging Center, Pennington Biomedical Research Center, Baton Rouge, LA, United States; 2 Neurology Department, University of California, Davis, Davis, CA, United States

**Keywords:** cognition, Alzheimer's disease, aging, neuroimaging, MRI

From a distance, the major science news events of the past few years might suggest that research on neuroimaging of the aging brain is becoming less important. In the United States at least, drugs that reduce levels of cerebral amyloid are available for a large number of older adults that meet criteria for Alzheimer's disease, and conventional PET scans are available to ensure they are eligible for treatment. Routine brain MRIs monitor these patients for side effects. In addition, blood tests that could theoretically render the eligibility PET scans obsolete are emerging, and work continues on blood tests that could also make the safety MRIs obsolete. On the prevention side, multiple large studies (Livingston et al., 2024), including U.S. POINTER (Baker et al., 2025) most recently, showed that a multicomponent lifestyle intervention is capable of slowing the rate of cognitive decline, relative to a control condition, among individuals at elevated risk of dementia. It is easy to see these exciting developments from a distance and conclude that we now understand how to prevent and treat dementia using well-understood and conventional tools, and therefore there is no more aging-related neuroimaging research to do.

As the authors of articles in this Research Topic might say: Not so fast.

For example, while U.S. POINTER and its peer interventions promote increases in physical activity and modification of the diet, our understanding of the biological mechanisms through which diet and exercise promote better cognitive function is rudimentary at best. In this Research Topic, Smith et al. seeks to increase our understanding of these mechanisms by reporting that among older adults, greater cardiorespiratory fitness (CRF) is related to better white matter integrity (WMI) in specific axon tracts. They suggested that higher CRF may help promote the maintenance of myelin within those tracts, with downstream benefits for cognitive functions that rely on those tracts. Karavasilis et al. reported that higher adherence to a Mediterranean dietary pattern was associated with resting-state functional connectivity (FC) in specific brain networks and, interestingly, that FC in these same networks associated with better cognitive function in specific domains, but only among participants with high adherence. Together, these two articles delineate brain mechanisms by which health-related lifestyles can support healthy cognitive trajectories in older adults and advance our knowledge of both structural and functional bases for possible lifestyle behavior effects on cognition.

In addition, while the health behaviors addressed by U.S. POINTER are known to have effects on cognitive function as we age, and while there are additional risk factors that can increase risk of dementia in a broad sense, a large amount of the variability in age-related cognitive change is poorly explained in terms of these factors. Several articles in this Research Topic explore young-old differences in neuroimaging characteristics (Bethlehem et al., 2022; Setton et al., 2023; Spreng et al., 2010; Meunier et al., 2014) in a search for a better understanding for why some older adults face such a precipitous drop in cognition while others remain well preserved. Miura et al. explored motor task brain activation differences in ipsilateral and contralateral cortical regions. They found that ipsilateral activations in dorsal premotor cortex among young participants was associated with greater dexterity, but there was no such association between activation and dexterity among older participants, whose dexterity was also reduced on average. It is possible that the ipsilateral activations are aiding task dexterity by inhibiting noisy contralateral activations while improving the coupling of the relevant contralateral activations, but this mechanism becomes disrupted in older adults. Lu et al. examined age differences in activation to a visual perception task using functional near-infrared spectroscopy (fNIRS). They found that in young participants, task activations were strongly unilateral, but were mainly bilateral among older individuals, consistent with hypotheses of age-related dedifferentiation or compensation. In another fNIRS study, Ćurčić-Blake et al. found that although performance on tasks of working memory and verbal fluency did not differ between Okinawan and Dutch participants, the Okinawans showed less activation in task-relevant brain areas, perhaps hinting at improved brain resource efficiency among these residents of a high-longevity “blue zone” (Pes et al., 2022). Finally, Taimouri and Ravindra estimated signatures of functional connectivity that were individual-specific and constant across age. In the context of aging, these findings suggest that functional activation and connectivity remodeling could be one vehicle through which the brain adapts—or fails to adapt—to the rigors of aging.

Returning to the topic of exciting new anti-amyloid treatments: ironically, these treatments have placed a spotlight on the urgent need to understand an entirely different biological substrate for brain aging—cerebrovascular distress—since the most serious side effects of those treatments are vascular in nature. In this Research Topic, Mohammadi et al. used phase-contrast MRI and NIRS in older adults to determine that greater interhemispheric differences in cerebral pulsatility index (PI) were associated with greater interhemispheric differences in Stroop task-evoked oxyhemoglobin concentration changes, but only among older adults. This result suggests that aging affects cerebral pulsatility, which in turn might drive functional reorganization of the brain. Zeng et al. measured PI and wall shear stress (WSS) of the carotid artery and computed neurovascular coupling (NVC) as the voxelwise correlation between cerebral blood flow and amplitude of low frequency fluctuation. They found that PI was elevated, while WSS and NVC were reduced, in individuals with cerebral small vessel disease (CSVD) compared to controls. Reduced WSS was associated with lower NVC, but surprisingly, so was reduced PI. The authors theorized that regionally sensitive changes in PI may occur in early CSVD, possibly having a compensatory effect of sustaining NVC that outweighed the detriments of increased arterial wall stress. Thammasart et al. characterized relative cerebral blood flow (rCBF) in white matter hyperintensities (WMH) and surrounding tissue, to assess how rCBF might influence the progression of WMH. They found lower rCBF in the lesions themselves compared to other tissue types with lesions located near the ventricles showing the largest reductions. They further observed that rCBF at baseline was lower in lesions that increased in size over 2 years compared to those that remained stagnant. Finally, Zhang J. et al. studied interhemispheric functional connectivity in patients with basal ganglia ischemic stroke (BGIS) and healthy controls using voxel-mirrored homotopic connectivity (VMHC) measured from resting-state functional magnetic resonance imaging (rs-fMRI). The authors found that individuals with unilateral BGIS had lower VMHC than controls, suggesting reduced synchronization and coordination between the left and right hemispheres. These articles add pieces to an increasingly complex vascular brain aging puzzle which involves blood flow dynamics, blood vessel structure, interactions between vascular and brain tissues, and downstream effects on cognition.

Given the predominance of brain MRI markers associated with specific clinical conditions such as Alzheimer's disease, it might appear that we have a full and complete understanding of brain-behavior relationships but this is not true either. In this Research Topic, Langer et al. used task-based fMRI to illustrate differing patterns of intra-network connectivity associated with semantic or rhyming decisions as well as differences in between-network connectivity, especially during the rhyming blocks. Putra et al. used MRI-based regional gray matter volumes to predict which older drivers are at risk for reduced driving safety performance, with mixed success. Finally, Wang et al. used fNIRS to show that among those with normal hearing, brain signal variability increased with increasing signal-to-noise (SNR) loads and correlated with performance on the task. However, hearing loss reduced brain signal variability, especially in noisy settings, and among those with hearing loss, brain signal variability was only correlated with performance in one of the SNR conditions.

Finally, the aforementioned recent research advances are specific to Alzheimer's disease (AD), and unfortunately there are multiple additional maladies of aging brain that are less well understood on a biological level. Binswager's disease (BD), for example, is a type of cerebral small vessel disease (Litak et al., 2020) often leading to increased risk of strokes and gradual cognitive impairment. Zhang H. et al. used multiparameter rs-fMRI to determine that participants with BD and mild cognitive impairment (MCI) showed reduced connectivity in specific brain functional networks, suggesting that reduced coordination among these networks may play a critical role in early cognitive decline. Multiple system atrophy (MSA) is a rare, fatal neurodegenerative disease, mainly presenting motor symptoms and decreased autonomic function (Fanciulli and Wenning, 2015). Cognitive impairment may or may not occur. Chen B. et al. used ^18^F-fluorodeoxyglucose (^18^F-FDG) to determine that reduced rates of glucose metabolism in the right superior frontal gyrus and right superior parietal lobule classified cognitively impaired vs. normal cognition groups with high accuracy, suggesting that this could be a useful biomarker for diagnosing cognitive impairment in MSA. Even within AD there is heterogeneity of biology (Winblad et al., 2016), with white matter abnormalities often but not always present (Ji et al., 2019). In a different study, Chen Y. et al. reported details of the progression of AD associated specifically with the presence of WMH. Comparing groups of AD participants with and without WMH presence, they found that the WMH group had reduced levels of functioning in several cognitive evaluation instruments, suggesting that WMH are correlated with increased cognitive decline and psychological symptoms among already cognitively impaired AD patients.

In summary, bystanders should not get the wrong idea. As these articles collectively point out, there is a great deal we do not understand about why the brain changes as it does during aging, why our health behaviors affect it the way they do, why certain brain characteristics exhibit themselves as cognitive symptoms while others do not, and why brain changes occur in some people but not others. Because of these unknowns, neuroimaging research into the aging brain will continue to be in high demand for decades to come.
